# Sociodemographic and Occupational Factors Associated With COVID-19 Vaccine and Influenza Vaccine Uptake Among Healthcare Workers, in Albania, 2022–2023: A Multicenter Study

**DOI:** 10.1093/cid/ciaf202

**Published:** 2025-06-03

**Authors:** Anisa Xhaferi, Silvia Bino, Rovena Daja, Adela Vasili, Jonilda Sulo, Nana Mebonia, Earta Ndreu, Miljana Nika, Nadire Jani, Ermiona Dabaj, Nensi Sustarova, Anila Moçi, Dorina Toçi, Albana Fico, Eugena Tomini, Sara Robinson, Pernille Jorgensen, Mark A Katz

**Affiliations:** Institute of Public Health, Tirana, Albania; Mediterranean and Black Sea Programme in Intervention Epidemiology Training (MediPIET), European Centre for Disease Prevention and Control (ECDC) fellow, Stockholm, Sweden; Institute of Public Health, Tirana, Albania; University of Medicine, Tirana; Tirana, Albania; South East European Center for Infectious Diseases Surveillance and Control, Tirana, Albania; Institute of Public Health, Tirana, Albania; Institute of Public Health, Tirana, Albania; Institute of Public Health, Tirana, Albania; Mediterranean and Black Sea Programme in Intervention Epidemiology Training (MediPIET), European Centre for Disease Prevention and Control (ECDC) fellow, Stockholm, Sweden; South East European Center for Infectious Diseases Surveillance and Control, Tirana, Albania; European Center for Disease Prevention and Control, Stockholm, Sweden; South East European Center for Infectious Diseases Surveillance and Control, Tirana, Albania; Mother Teresa University Hospital Tirana, Albania; Regional Hospital of Durres, Albania; Regional Hospital of Durres, Albania; Regional Hospital of Durres, Albania; Regional Hospital of Fier, Albania; Institute of Public Health, Tirana, Albania; University of Medicine, Tirana; Tirana, Albania; Mother Teresa University Hospital Tirana, Albania; Institute of Public Health, Tirana, Albania; University of Medicine, Tirana; Tirana, Albania; Maine Department of Health and Human Services, USA; World Health Organization, Regional Office for Europe, Copenhagen, Denmark; World Health Organization, Regional Office for Europe, Copenhagen, Denmark

**Keywords:** Albania, COVID-19, influenza, vaccination, health care workers

## Abstract

**Background:**

Healthcare workers (HCWs) are a priority group for coronavirus disease 2019 (COVID-19) and influenza vaccination. We evaluated sociodemographic and occupational factors, attitudes, and knowledge associated with the uptake of primary and booster doses of COVID-19 and seasonal influenza vaccines among HCWs.

**Methods:**

Between February 2022 and February 2023, we administered a structured questionnaire to HCWs in 3 Albanian hospitals who were enrolled in a multiyear cohort study. The questionnaire assessed participants' knowledge, confidence, and attitudes toward COVID-19 and seasonal influenza vaccines, and included questions on COVID-19 and influenza vaccination history, perceptions of vaccine safety and effectiveness, and factors influencing vaccine decision-making.

**Results:**

We included 1456 HCWs. Their median age was 44 (interquartile range: 33–53) and 77.3% were female. Overall, 20.7% were physicians, 47.0% nurses or midwives, and 21.5% support staff. In all, 93.6% received a COVID-19 primary vaccine series, 20% a COVID-19 booster, and 23.7% received an influenza vaccine in the 2022–2023 season. In the multivariable analysis, male HCWs were more likely to receive COVID-19 boosters (adjusted odds ratio [aOR] 2.08; 95% confidence interval [CI]: 1.55–2.80). However, they were less likely to receive influenza vaccines (aOR 0.69; 95% CI: .51–.95). Medical doctors were more likely to receive COVID-19 vaccine doses, while nurses had higher uptake of the influenza vaccine (aOR 1.87; 95% CI: 1.30–2.68). Confidence in the safety and effectivity of both vaccines was positively associated with vaccine uptake.

**Conclusions:**

Among HCWs, primary series COVID-19 vaccine coverage was high, but COVID-19 booster doses and seasonal influenza vaccines were low. Findings related to age and sex differences among HCW groups with low vaccine uptake, as well as their knowledge and attitudes toward vaccines, should inform targeted strategies to improve COVID-19 and influenza vaccine uptake, particularly among younger HCWs, female HCWs, and nonphysicians.

## Teaser

Among 1456 Albanian healthcare workers, COVID-19 primary vaccine series uptake was >90%, COVID-19 booster uptake was only 20%, and influenza vaccination uptake in 2022–2023 was 23%. Uptake was higher among healthcare workers who expressed confidence in vaccine safety and effectiveness.

In late 2019, the novel coronavirus known as severe acute respiratory syndrome coronavirus 2 (SARS-CoV-2) emerged. On 11 March 2020, the World Health Organization (WHO) declared coronavirus disease 2019 (COVID-19) a pandemic. The first case of COVID-19 in Albania was detected on 8 March 2020, and as of 26 May 2024, 334,863 cases and 3605 deaths had been reported in the country [1,2].

COVID-19 vaccination has been a critical intervention to reduce COVID-19–related morbidity and mortality. In the first year of the pandemic alone, COVID-19 vaccines were estimated to have saved 20 million lives globally [3].

Healthcare workers (HCWs) have been prioritized for COVID-19 vaccination since vaccines first became available. In November 2023, WHO's Strategic Advisory Group of Experts on Immunization reaffirmed the recommendation that HCWs remain a priority group for COVID-19 vaccination [4].

In Albania, an upper middle-income country, COVID-19 vaccination began on 11 January 2021. HCWs were prioritized for COVID-19 vaccination by the Albanian National Technical Advisory Group for Immunization and recommended to receive a 2-dose primary series. On 15 October 2021, the Albanian National Technical Advisory Group for Immunization recommended that all HCWs receive a booster dose (third dose) 6 months after their primary vaccine series and a fourth dose within 1 year after the last dose of the COVID-19 vaccine [5]. Current WHO guidelines recommend that HCWs with direct patient contact receive an up-to-date COVID-19 vaccine every 12 months [6].

Through 9 June 2024, COVID-19 vaccination coverage among HCWs in Albania was 95.9% for the first dose, 83.7% for the second dose, 11.8% for a third dose, and 8.2% for a fourth dose (unpublished data, Albania National Vaccination Program). These coverage levels were lower compared to COVID-19 vaccine uptake among HCWs enrolled in a multicenter study in 7 European Union countries [7]. In that study, as of autumn 2023, COVID-19 vaccine coverage was 2.3% for 2 doses, 12.7% for 3 doses, 44.7% for 4 doses, and 39.7% for 5 doses. In another study conducted in United States, during 2021–2022, COVID-19 vaccine coverage among HCWs was 87.3% for primary doses (2 doses) and 67.1% reported receiving a 4-COVID-19 booster dose [8].

Seasonal influenza is estimated to cause between 3 and 5 million cases of severe illness and 250 000–500 000 deaths globally each year [9]. WHO recommends annual influenza vaccination for HCWs to reduce influenza transmission, reduce morbidity and mortality, and reduce work absenteeism among HCWs [10]. Different studies around the world have shown the importance of the influenza vaccine in protecting HCWs [11–14]. In Albania, HCWs are recommended to get vaccinated annually against influenza, but influenza vaccination is not mandatory. During the 2022–2023 influenza season, 40% of all HCWs in Albania were vaccinated against influenza [15]. Influenza vaccine coverage level in Albania was comparable to the findings of a meta-analysis that included studies that reported influenza vaccine uptake data among HCWs in 26 countries across seven regions; that study reported a global influenza vaccination rate of 41.7% [16].

Although little is known about barriers to COVID-19 and influenza vaccine among HCWs, barriers to vaccination in HCWs have been described in other countries. A study conducted in Europe and Central Asia found that HCWs’ concerns about vaccine efficacy, effectiveness, and long-term safety were common barriers to accepting the COVID-19 vaccine [17]. A review that included data from 31 studies from across the world identified a number of barriers to vaccination. These barriers included sociodemographic factors, such as age and sex, occupation, and lack of information about vaccines [18]. Understanding the factors associated with uptake of COVID-19 and influenza vaccines among HCWs can help inform interventions to increase uptake of the 2 vaccines. A previous analysis evaluated COVID-19 vaccine uptake among HCWs during the 2021–2022 winter season in Albania [19].

In this study, we aimed to use more recent data for the 2022–2023 winter season to provide an updated analysis of the sociodemographic, occupational, and clinical factors associated with uptake of primary vaccine series and booster dose of COVID-19 and uptake of influenza vaccine during the 2022–2023 winter season. In addition, we evaluated knowledge, attitudes, and practices associated with COVID-19 and influenza vaccine uptake.

## METHODS

### Study Design and Population

We analyzed data collected between February 2022 and February 2023 from an ongoing prospective longitudinal cohort study on COVID-19 and influenza among HCWs in 3 hospitals of Albania, which began in February 2021. The cohort study methods have been described previously [20].

Briefly, we offered enrollment in the study to all HCWs in the 3 Albanian hospitals. These hospitals were selected because they each employ a large number of HCWs, and they are centrally located, facilitating sample transport to the centrally located national Institute of Public Health laboratories. In 2021, all HCWs eligible for COVID-19 vaccination in the 3 hospitals were invited to join the study [20]. HCWs included all categories of hospital staff, both clinical and nonclinical, including the administration sector. Participants did not receive COVID-19 or influenza vaccine as part of the study, and participation was voluntary. While the COVID-19 primary vaccine series (PVS) was mandatory for all HCWs working on Health Care Systems during 2021–2022, COVID-19 boosters and influenza vaccines were recommended but not mandatory. HCWs could only avoid vaccination by providing justification for contraindications related to allergies; otherwise, they had to pause their work. All participants who received only 1 dose of the COVID-19 vaccine did so by choice; HCWs were not advised to limit their vaccination to a single dose. At enrollment (2021), and then again during the second year of the study (February–June 2022), participants completed a questionnaire with questions about demographics, occupation, and knowledge, attitudes, and practices about COVID-19 and influenza vaccines.

During the study, participants completed weekly questionnaires, where they were asked whether they had recently received a COVID-19 vaccine. From September 2022 to February 2023, as part of the weekly questionnaires, participants were also asked whether they had received an influenza vaccine in the 2022–2023 influenza season. Study staff confirmed participants' COVID-19 and influenza vaccination history using the National Vaccination System database [21, 22]. Discrepancies between self-report and registry data were resolved through follow-up with study participants. Study data were uploaded and stored using REDCap [23].

### Statistical Analysis

For the COVID-19 vaccine, we assessed participants’ vaccination status as of 27 February 2023. For the influenza vaccine, we considered an individual vaccinated for the 2022–2023 season if they had received an influenza vaccine between 1 September 2022 and 27 February 2023.

We conducted a descriptive analysis to detail the frequency of sociodemographic variables, underlying conditions, and previous SARS-CoV-2 infection with COVID-19 among HCWs who had received COVID-19 primary vaccine series, COVID-19 booster doses, and influenza vaccine. Primary exposures of interest were age, sex, self-reported health status, self-reported preexisting chronic conditions (eg, cardiac disease, hypertension, diabetes), profession, smoking habits, household size, patient care responsibilities, and knowledge and attitudes about the safety and effectiveness of the vaccines.

We used uni- and multivariable logistic regression analyses to identify factors independently associated with receiving COVID-19 primary series (2 doses) compared to being unvaccinated, factors associated with receiving primary vaccine series, and at least 1 COVID-19 booster vaccine (3 doses or more) compared to those who received a primary vaccine series (2 doses), and factors associated with receiving the 2022–2023 influenza vaccine compared to those who were unvaccinated against influenza in that season.

We excluded participants from the COVID-19 vaccine analysis if they had received only 1 dose of COVID-19 vaccine. Participants who dropped out during the course of the 12-month follow-up were included in all of the analyses. We referred to their vaccination status at the time they withdrew from the study. All the questionnaires were completed in full, so we did not address issues of missing data.

In the multivariable logistic regression, we included all variables potentially related to the outcome that reached a significance level of *P* < .05 in the univariable analyses.

The most significant factors associated with vaccination status were identified using backward stepwise eliminationto ensure that all variables were initially considered. This approach is efficient in identifying the most parsimonious model while controlling for potential confounders. Using this method, we removed the least significant variables (those with the highest *P* values) 1 by 1. Crude odds ratios and adjusted odds ratios (aOR), accounting for potential confounders, were calculated with 95% confidence intervals (CI) for each association to ensure robust and interpretable results.

All analyses were performed using IBM SPSS Statistics 25 [24].

## RESULTS

### Characterization of Study Participants

As of 27 February 2023, 1456 HCWs from 3 hospitals in Albania were enrolled in the study, representing approximately 39% of all HCWs in these hospitals. The majority of participants were from Tirana Mother Teresa University Hospital (62.2%), followed by Durres Regional Hospital (20.1%) and Fier Regional Hospital (17.7%). The median age of participants was 44 years (range: 22–71 years; interquartile range: 33–53). Most participants were female (1126, 77.3%). The primary occupations were nurses or midwives (985, 67.7%) and medical doctors (301, 20.7%) (Table 1).

**Table 1.** Sociodemographic, Occupational, and Clinical Characteristics Associated With at Least Two Doses (Primary Vaccine Series) of COVID-19 Vaccine Among Albanian HCWs 2022–2023

	COVID-19 Vaccination Status		OR			
Sociodemographic, occupational, clinical characteristics	Unvaccinated N (%)	PVS^a^ or More N (%)	OR (95% CI)	*P* Value	aOR (95% CI)	*P* Value
Total	N = 93 (100)	N = 1322 (100)				
*Hospital study site*						
Mother Teresa University Hospital Tirana	60 (6.8)	822 (93.2)	Ref	…	Ref	
Regional Hospital of Durres	10 (3.5)	278 (96.5)	2.03 (1.03–4.02)	.042	1.89 (.95–3.77)	.070
Regional Hospital of Fier	23 (9.4)	222 (90.6)	0.71 (.43–1.17)	.172	0.72 (.43–1.20)	.210
*Age group (y)*						
≤34	42 (11.4)	326 (88.6)	Ref	…	Ref	
35–44	23 (6.8)	316 (93.2)	1.77 (1.04–3.01)	.035	1.72 (1.00–2.95)	.049
45–54	10 (2.5)	395 (97.5)	5.09 (2.51–10.3)	<.001	4.89 (2.40–9.97)	<.001
≥55	18 (5.9)	285 (94.1)	2.04 (1.15–3.62)	.015	1.98 (1.10–3.56)	.022
*Sex*						
Female	82 (7.5)	1012 (92.5)	Ref	…	Ref	
Male	11 (3.4)	310 (96.6)	2.28 (1.20–4.34)	.012	2.26 (1.15–4.45)	.018
*Occupation*						
Medical doctor	12 (4.1)	282 (95.9)	Ref	…	Ref	
Nurse, midwife	43 (6.4)	628 (93.6)	.62 (.32–1.20)	.155	0.87(.44–1.70)	.681
Support staff	20 (6.6)	284 (93.4)	0.60 (.29–1.26)	.179	0.71 (.34–1.50)	.368
Administrative staff	18 (12.3)	128 (87.7)	0.30 (.14–.65)	.002	0.34 (.16–.75)	.007
*Provides hands-on medical care to patients*						
No	40 (7.1)	521 (92.9)	Ref	…	…	
Yes	53 (6.2)	801 (93.8)	1.16 (.76–1.78)	.493	…	…
*Household size*						
Living alone	2 (9.1)	20 (90.9)	Ref	…	…	
Living with 1–2	34 (6.6)	478 (93.4)	1.41 (.32–6.27)	.655	…	…
Living with 3–5	54 (6.5)	774 (93.5)	1.43 (.33–6.29)	.633	…	…
Living with 6 or more	3 (5.7)	50 (94.3)	1.67 (.26–10.74)	.591	…	…
*Underlying conditions*						
No chronic condition	79 (6.5)	1131 (93.5)	Ref	…	…	
One or more chronic conditions	14 (6.8)	191 (93.2)	0.95 (.53–1.72)	.873	…	…
Current smoking history						
Never smoked	89 (6.9)	1193 (93.1)	Ref	…	…	
Current or former smoker	4 (3.0)	129 (97.0)	2.41 (.87–6.66)	.091	…	…
*Positive laboratory test previous for SARS-CoV-2 (before vaccination)*						
No	54 (5.9)	864 (94.1)	Ref	…	…	
Yes	39 (7.8)	458 (92.2)	0.73 (.48–1.13)	.156	…	…
*Self-rated health status*						
Poor	2 (7.4)	25 (92.6)	Ref	…	…	
Good	91 (6.6)	1297 (93.4)	1.14 (.27–4.89)	.860	…	…
*Having extensive knowledge about the COVID-19 vaccine*						
Some	80 (7.2)	1030 (92.8)	Ref	…	…	
A lot	13 (4.3)	292 (95.7)	1.75 (.96–3.18)	.069	…	…
*COVID-19 vaccination is safe*						
Disagree	53 (19.3)	222 (80.7)	Ref	…	Ref	
Agree	40 (3.5)	1100 (96.5)	6.57 (4.25–10.14)	<.001	6.63 (4.22–10.22)	<.001
*COVID-19 vaccination is effective*						
Not effective	55 (12.7)	378 (87.3)	Ref	…	Ref	
Effective	38 (3.9)	944 (96.1)	3.62 (2.23–5.56)	<.001	3.48 (2.25–5.39)	<.001
*Worried are you about getting sick with COVID-19 during the next 12 mo*						
Worried	55 (7.1)	724 (92.9)	Ref	…	…	
Not worried	38 (6.0)	598 (94.0)	1.20 (.78–1.83)	.413	…	…
If I am fully vaccinated against COVID-19, I will be less likely to miss work because of getting sick with COVID-19						
Disagree	10 (4.7)	203 (95.3)	Ref	…	Ref	…
Agree	83 (6.9)	1119 (93.1)	0.66 (.34–1.30)	.233		

Abbreviations: aOR, adjusted odds ratio; CI, confidence interval; COVID-19, coronavirus disease 2019; PVS, primary vaccine series; OR, odds ratio; SARS, CoV-2, severe acute respiratory syndrome coronavirus 2.

^a^Primary vaccine series (2 doses) including individuals who may have received additional doses.

Most HCWs (875, 60.1%) reported providing direct medical care to patients. Most participants (1388, 98.1%) self-rated their health status as good, while 205 (14.5%) reported having at least 1 chronic medical condition. Overall, 133 (9.4%) participants identified as current or former smokers. Most participants believed that the COVID-19 vaccine was safe (1140, 80.6%) and effective (982, 69.4%). In contrast, fewer than half of participants said that influenza vaccine was safe (710, 48.8%) and effective (529, 36.3%). During the 12-month study period, 75 participants dropped out the study.

### COVID-19 and Influenza Vaccine Coverage Among Study Participants

A total of 1050 participants (72.1%) received 2 COVID-19 vaccine doses (PVS), 271 participants (18.6%) received 3 doses (PVS plus a booster dose), only 1 participant (∼0.1%) received a fourth vaccine dose and 93 (6.4%) participants remained unvaccinated. For PVS, most HCWs (913, 87%) HCWs received Pfizer-BioNTech, and of the 272 booster doses, nearly all (259, 95.2%) were Pfizer-BioNTech. Overall (41, 2.8%), participants received only 1 dose and were excluded from the analysis (Table 1).

Fewer than one-fourth of the participants (345, 23.7%) received an influenza vaccine during the 2022–2023 influenza season. Of those vaccinated, the majority (302, 87.5%) received GC FLU Quadrivalent vaccine from Green Cross Corporation; 49 (14.2%) received Sanofi vaccine (Vaxigrip or Vaxigrip Tetra). Nearly all HCWs vaccinated against influenza received their vaccine at the workplace, while 43 participants (12.5%) were vaccinated in a private setting. More than half of the participants (813, 55.8%), were not worried about getting sick with influenza in the next 12 months. Additionally, most participants (1268, 87.1%), disagreed with the statement “If I am fully vaccinated against influenza, I will be less likely to miss work because of getting sick with influenza” (Table 3).

### Factors Associated With Receiving Primary Vaccine Series of COVID-19 Vaccine

In the multivariable analysis, factors positively associated with receiving COVID-19 PVS (*P* < .05) were: being in age groups 35–44 years (aOR 1.72; 95% CI: 1.00–2.95), 45–54 years (aOR 4.89; 95% CI: 2.40–9.97), >55 years (aOR 1.99; 95% CI: 1.10–3.56) compared to HCWs ≤34 years old. In addition, being male (aOR 2.26; 95% CI: 1.15–4.45) compared with female, confidence in the safety of COVID-19 vaccines (aOR 6.63; 95% CI: 4.22–10.22) and confidence in the effectiveness of COVID-19 vaccines (aOR 3.48; 95% CI: 2.25–5.39) were all positively associated with receiving COVID-19 PVS. Conversely, being administrative staff was associated with lower odds of receiving primary doses of the COVID-19 vaccine compared to being a medical doctor (aOR 0.34; 95% CI: .16–.75) (Table 1).

### Factors Associated With Receiving Booster Doses of COVID-19 Vaccine

In the multivariable analysis, several factors were positively associated with the uptake of booster doses. Compared with HCWs ≤34 years old, HCWs 35–44 years old (aOR 1.69; 95% CI: 1.07–2.66), 45–54 years old (aOR 2.81; 95% CI: 1.85–4.25); and ≥55 years old (aOR 2.74; 95% CI: 1.77–4.25) were more likely to have received a booster dose. In addition, being male (aOR 2.08; 95% CI: 1.55–2.80) compared with female, having direct contact with patients (aOR 1.56; 95% CI: 1.17–2.09), having extensive knowledge about the COVID-19 vaccine (aOR 1.44; 95% CI: 1.02–2.03), confidence in the safety of COVID-19 vaccines (aOR 2.71; 95% CI: 1.62–4.53) and confidence in the effectivity of COVID-19 vaccines (aOR 2.70; 95% CI: 1.84–3.99) were all positively associated with booster vaccine receipt.

In contrast, certain occupational roles were associated with lower odds of receiving a booster. Compared with medical doctors, nurse and midwives (aOR 0.30; 95% CI: .21–.42), support staff (aOR 0.16; 95% CI: .10–.25), and administrative staff (aOR 0.13; 95% CI: .07–.25) had lower odds of receiving a booster dose. Additionally, HCWs at Durres Regional Hospital (aOR 0.64; 95% CI: .44–.93) and Fier Regional Hospital (aOR 0.36; 95% CI: .22–.60) had lower odds of receiving COVID-19 vaccine booster doses compared with HCWs at Mother Teresa University Hospital, Tirana (Table 2).

**Table 2.** Sociodemographic, Occupational, and Clinical Characteristics Associated With Uptake of COVID-19 Booster Doses Among Albanian HCWs, Compared to Those Who Received Only Primary COVID-19 Vaccine Series, 2022–2023

Sociodemographic, Occupational, Clinical Characteristics	COVID-19 Vaccination Status		OR			
	Vaccinated With Only 2 Doses	Vaccinated With Booster Doses	OR (95% CI)	*P* Value	aOR (95% CI)	*P* Value
Total	N = 1050 (100)	N = 272 (100)				
*Hospital study site*						
Mother Teresa University Hospital Tirana	625 (76.0)	197 (24.0)	Ref	…	Ref	
Regional Hospital of Durres	225 (80.9)	53 (19.1)	0.75 (.53–1.05)	.093	0.64 (.44–.93)	.020
Regional Hospital of Fier	200 (90.1)	22 (9.9)	0.35 (.22–.56)	<.001	0.36 (.22–.60)	<.001
*Age group (y)*						
≤34	290 (89.0)	36 (11.0)	Ref	…	Ref	
35–44	260 (82.3)	56 (17.7)	1.74 (1.11–2.72)	.017	1.69 (1.07–2.66)	.024
45–54	290 (73.4)	105 (26.6)	2.92 (1.93–4.40)	<.001	2.81 (1.85–4.25)	<.001
≥55	210 (73.7)	75 (26.3)	2.88 (1.86–4.45)	<.001	2.74 (1.77–4.25)	<.001
*Sex*						
Female	837 (82.7)	175 (17.3)	Ref	…	Ref	
Male	213 (68.7)	97 (31.3)	2.18 (1.63–2.91)	<.001	2.08 (1.55–2.80)	<.001
*Occupation*						
Medical doctor	157 (55.7)	125 (44.3)	Ref	…	Ref	
Nurse, midwife	524 (83.4)	104 (16.6)	0.25 (.18–.34)	<.001	0.30 (.21–.42)	<.001
Support staff	253 (89.1)	31 (10.9)	0.15 (.10–.24)	<.001	0.16 (.10–.25)	<.001
Administrative staff	116 (90.6)	12 (9.4)	0.13 (.07–.25)	<.001	0.13 (.07–.25)	<.001
*Provides hands-on medical care to patients*						
No	429 (82.3)	92 (17.7)	Ref	…	Ref	
Yes	621 (77.5)	180 (22.5)	1.35 (1.02–1.79)	.035	1.56 (1.17–2.09)	.003
*Household size*						
Living alone	7 (35.0)	13 (65.0)	Ref	…	Ref	
Living with 1–2	370 (77.4)	108 (22.6)	0.16 (.06–.40)	<.001	0.31 (.22–.44)	<.001
Living with 3–5	633 (81.8)	141 (18.2)	0.12 (.05–.31)	<.001	0.16 (.10–.25)	<.001
Living with 6 or more	40 (80.0)	10 (20.0)	0.14 (.04–.43)	.001	0.13 (.07–.25)	<.001
*Underlying conditions*						
No chronic condition	912 (80.6)	219 (19.4)	Ref	…	Ref	
One or more chronic conditions	138 (72.3)	53 (27.7)	1.60 (1.13–2.27)	.008	1.28 (.87–1.90)	.215
*Current smoking history*						
Never smoked	958 (80.3)	235 (19.7)	Ref	…	Ref	
Current or former smoker	92 (71.3)	37 (28.7)	1.64 (1.09–2.46)	.017	1.18 (.75–1.83)	.476
*Positive laboratory test previous for SARS-CoV-2*						
No	687 (79.5)	177 (20.5)	Ref	…	…	
Yes	363 (79.3)	95 (20.7)	1.02 (.77–1.34)	.913	…	…
*Self-rated health status*						
Poor	19 (76.0)	6 (24.0)	Ref	…	…	
Good	1031 (79.5)	266 (20.5)	1.22 (.48–3.10)	.669	…	…
*Having extensive knowledge about the COVID-19 vaccine*						
Some	861 (83.6)	169 (16.4)	Ref	…	Ref	
A lot	189 (64.7)	103 (35.3)	2.78 (2.08–3.72)	<.001	1.44 (1.02–2.03)	.037
*COVID-19 vaccination is safe*						
Disagree	204 (91.9)	18 (8.1)	Ref	…	Ref	
Agree	846 (76.9)	254 (23.1)	3.40 (2.06–5.62)	<.001	2.71 (1.62–4.53)	<.001
*COVID-19 vaccination is effective*						
Not effective	342 (90.5)	36 (9.5)	Ref	…	Ref	
Effective	708 (75.0)	236 (25.0)	3.17 (2.18–4.60)	<.001	2.71 (1.84–3.99)	<.001
*Worried are you about getting sick with COVID-19 during the next 12 mo*						
Worried	579 (80.0)	145 (20.0)	Ref	…	…	
Not worried	471 (78.8)	127 (21.2)	0.93 (.71–1.21)	.588	…	…
If I am fully vaccinated against COVID-19, I will be less likely to miss work because of getting sick with COVID-19						
Disagree	165 (81.3)	38 (18.7)	Ref	…	Ref	…
Agree	885 (79.1)	234 (20.9)	1.15 (.78–1.68)	.477	…	…

Abbreviations: aOR, adjusted odds ratio; CI, confidence interval; COVID-19, coronavirus disease 2019; HCW, healthcare worker; OR, odds ratio; SARS, CoV-2, severe acute respiratory syndrome coronavirus 2.

### Factors Associated With Receiving Influenza Vaccine

In the multivariable analysis, several factors were positively associated with receiving influenza vaccine. HCWs aged 45–54 years old (aOR 1.59; 95% CI: 1.14–2.23) and >55 years old (aOR 1.47; 95% CI: 1.02–2.10) were more likely to have received influenza vaccine compared to those ≤34 years old. In addition, nurse and midwives (aOR 1.87; 95% CI: 1.30–2.68) and support staff (aOR 1.53; 95% CI: 1.02–2.30) were more likely to have received influenza vaccine compared to medical doctors. Likewise, confidence in the safety of influenza vaccines (aOR 2.15; 95% CI: 1.68–2.77), confidence in the effectivity of influenza vaccines (aOR 1.64; 95% CI: 1.28–2.10), and agreeing that being vaccinated against influenza would make one less likely to miss work (aOR 2.28; 95% CI: 1.64–3.15) were all positively associated with receiving influenza vaccine. Finally, HCWs at Fier Regional Hospital were significantly more likely to have received the influenza vaccine compared to those at Mother Teresa University Hospital (aOR 5.90; 95% CI: 4.35–7.99), while HCWs at Durres Regional Hospital were less likely to have received the influenza vaccine compared to those working at Mother Teresa University Hospital (aOR 0.60; 95% CI: .41–.89).

In contrast, males (aOR 0.69; 95% CI: .51–.95) and HCWs at Durres Regional Hospital (compared to HCWs at Mother Teresa University Hospital) (aOR 0.61; 95% CI: .41–.90) had lower odds of receiving influenza vaccine (Table 3).

**Table 3.** Sociodemographic, Occupational, and Clinical Characteristics of Albanian HCWs and Attitudes and Practices Associated With Influenza Vaccine Uptake Among HCWs, 2022–2023

Characteristics	Influenza Vaccination Status		OR			
	Unvaccinated	Vaccinated With Flu Vaccine in 2022–2023 season	OR (95% CI)	*P* Value	aOR (95% CI)	*P* Value
Total	N = 1111 (76.3%)	N = 345 (23.7%)				
Hospital study site						
Mother Teresa University Hospital Tirana	739 (81.7)	166 (18.3)	Ref	…	Ref	
Regional Hospital of Durres	258 (88.1)	35 (11.9)	0.60 (.41–.89)	.012	0.60 (.41–.89)	.011
Regional Hospital of Fier	114 (44.2)	144 (55.8)	5.62 (4.18–7.57)	<.001	5.90 (4.35–7.99)	<.001
Age group, y						
≤34	315 (80.8)	75 (19.2)	Ref	…	Ref	
35–44	266 (76.4)	82 (23.6)	1.30 (.91–1.84)	.152	1.31 (.92–1.87)	.132
45–54	299 (73.1)	110 (26.9)	1.55 (1.11–2.16)	.011	1.59 (1.14–2.23)	.006
≥55	231 (74.8)	78 (25.2)	1.42 (.99–2.03)	.057	1.47 (1.02–2.10)	.038
Sex						
Female	845 (75.0)	281 (25.0)	Ref	…	Ref	
Male	266 (80.6)	64 (19.4)	0.72 (.53–.98)	.037	0.69 (.51–.95)	.021
Occupation						
Medical doctor	251 (83.4)	50 (16.6)	Ref	…	Ref	
Nurse, midwife	500 (73.1)	184 (26.9)	1.85 (1.31–2.62)	.001	1.87 (1.30–2.68)	.001
Support staff	238 (76.0)	75 (24.0)	1.58 (1.06–2.39)	.024	1.53 (1.02–2.30)	.041
Administrative staff	122 (77.2)	36 (22.8)	1.48 (.92–2.39)	.109	1.53 (.95–2.01)	.084
Provides hands-on medical care to patients						
No	453 (78.0)	128 (22.0)	Ref	…	…	
Yes	658 (75.2)	217 (24.8)	1.17 (.91–1.50)	.224	…	…
Household size						
Living alone	19 (86.4)	3 (13.6)	Ref	…	…	
Living with 1–2	396 (74.6)	135 (25.4)	2.16 (.63–7.41)	.221	…	…
Living with 3–5	659 (77.7)	189 (22.3)	1.82 (.53–6.20)	.341	…	…
6 or more	37 (67.3)	18 (32.7)	3.08 (.81–11.77)	.100	…	…
Underlying condition						
No chronic condition	957 (76.8)	289 (23.2)	Ref	…	…	
One or more chronic conditions	154 (73.3)	56 (26.7)	1.20 (.86–1.68)	.274	…	…
Current smoking history						
Never smoked	999 (75.6)	322 (24.4)	Ref	…	…	
Current or former smoker	112 (83.0)	23 (17.0)	0.64 (.40–1.02)	.059	…	…
Previous SARS-CoV-2 infection						
No	725 (77.1)	215 (22.9)	Ref	…	…	
Yes	386 (74.8)	130 (25.2)	1.14 (.88–1.46)	.319	…	…
Self-rated health status						
Poor	22 (81.5)	5 (18.5)	Ref	…	…	
Good	1089 (76.2)	340 (23.8)	1.37 (.52–3.66)	.525	…	…
Influenza vaccine is safe						
Disagree	619 (83.0)	127 (17.0)	Ref	…	Ref	
Agree	492 (69.3)	218 (30.7)	2.16 (1.68–2.77)	<.001	2.15 (1.68–2.77)	<.001
Influenza vaccine is effective						
Not effective	739 (79.7)	188 (20.3)	Ref	…	Ref	
Effective	372 (70.3)	157 (29.7)	1.66 (1.30–2.12)	<.001	1.64 (1.28–2.10)	<.001
Are you worried about getting sick with influenza during the next 12 mo						
Worried	496 (77.1)	147 (22.9)	Ref	…	…	
Not worried	615 (75.6)	198 (24.4)	1.09 (.85–1.39)	.506	…	…
If I am fully vaccinated against influenza, I will be less likely to miss work because of getting sick with influenza						
Disagree	997 (78.6)	271 (21.4)	Ref	…	Ref	
Agree	114 (60.6)	74 (39.4)	2.39 (1.73–3.30)	<.001	2.28 (1.64–3.15)	<.001
COVID-19 vaccination status						
Number of unvaccinated	68 (73.1)	25 (26.9)	Ref	…	…	
Number of vaccinated with primary series	830 (76.2)	261 (23.9)	.86 (.53–1.38)	.523	…	…
Number of vaccinated with primary series and booster	213 (78.3)	59 (21.7)	0.75 (.44–1.30)	.306	…	…

Abbreviations: aOR, adjusted odds ratio; CI, confidence interval; COVID-19, coronavirus disease 2019; HCW, healthcare worker; OR, odds ratio; SARS, CoV-2, severe acute respiratory syndrome coronavirus 2.

## DISCUSSION

In our study of factors associated with COVID-19 and influenza vaccine uptake among HCWs in Albania, we found that in early 2023 primary vaccination against COVID-19 was very high (93.6%) but only 20% of HCWs had received a COVID-19 booster, despite national recommendations that were in place since November 2021 for HCWs to receive a booster vaccination [25]. In addition, less than one quarter of HCWs received influenza vaccination during the 2022–2023 season. HCWs who believed vaccines were safe and effective were more likely have received COVID-19 vaccines and influenza vaccines, and older HCWs were more likely to have received both vaccines compared to younger HCWs. Our findings provide a framework to better tailor future COVID-19 and influenza vaccine promotional campaigns among HCWs in Albania.

We found that COVID-19 PVS and booster coverage rates among Albanian HCWs were largely unchanged compared to our previous analysis of the same cohort through 11 June 2022, highlighting that very few HCWs received an additional dose of COVID-19 vaccine from mid-2022 to early 2023 [19].

We found that among Albanian HCWs, concerns about safety and effectiveness of the vaccine were associated with lower uptake of both COVID-19 and influenza vaccine. This finding aligns with similar studies conducted globally, including a comprehensive worldwide assessment published in 2021 that examined COVID-19 vaccine hesitancy among HCWs [26, 27]. In this assessment, safety and effectiveness concerns were also identified as key barriers to vaccine uptake. This association was also observed recent studies conducted in the United States, including Puerto Rico [28]. Future vaccination campaigns should highlight evidence supporting the safety and effectiveness of both vaccines, for which there is abundant evidence and stress that evidence supports that the benefits outweigh risks for influenza and COVID-19 vaccine in target groups for vaccination like HCWs [29–31].

In our study, male HCWs were more likely to have received a COVID-19 vaccine booster compared to female HCWs. This finding is consistent with other studies conducted in United States, China, and Egypt, where male HCWs were also shown to have higher rates of COVID-19 vaccine compared to female HCWs [32–34]. Lower rates of COVID-19 vaccination among women may in part be attributable to concerns about how COVID-19 vaccines could affect and interact with conception, pregnancy, and breast feeding [35–37]. Such concerns have been identified particularly among younger women [38, 39]. However, studies confirm COVID-19 vaccines' safety and effectiveness in women, with the WHO recommending their use during pregnancy to reduce complications and infant morbidity [40–42].

In our study, younger HCWs (aged ≤34 years) were less likely to get vaccinated against both COVID-19 and influenza compared to those HCWs older than age 35 years. This trend of higher vaccine acceptance with increasing age could be due to higher perceived vulnerability among older adults, a perception supported by evidence from numerous COVID-19 studies worldwide. Additionally these findings could also be related to reproductive concerns among younger HCWs [43, 44]. Younger HCWs are still at risk of infection and severe disease from SARS-CoV-2 infection, and infected younger HCWs could transmit the virus to vulnerable patients if they work while infected. These points could be highlighted in future COVID-19 vaccination campaigns.

Physicians in our study were significantly more likely to receive a COVID-19 booster dose than nonphysicians. This trend aligns with findings from a global analysis published in 2022, which reviewed data from 48 studies worldwide. Similar to our study, our study found that physicians were significantly more likely to receive a COVID-19 booster compared to professionals in other healthcare roles [45, 46]. Conversely, in our study, nurses, midwives, and support staff were more likely to have been vaccinated against influenza compared with physicians, a finding that has been observed in previous years in Albania [47]. Similar findings were reported in the United Kingdom during the 2022–2023 season, where nurses in general practices had higher influenza vaccine uptake than other professionally qualified clinical staff [48]. Further studies should attempt to better understand differences in vaccine uptake by occupation among Albanian HCWs.

We found that less than one quarter of HCWs in Albania were vaccinated against influenza in the 2022–2023 season. This low uptake mirrors global challenges, where influenza vaccination rates among HCWs are also suboptimal. A meta-analysis showed that the overall vaccination rate against influenza among HCWs in 26 countries worldwide was 41.7%, underscoring the importance of improved influenza vaccination promotion campaigns among this population [16].

In contrast to COVID-19 vaccines, we found that influenza vaccine uptake was higher among females compared to males in our study, a finding that has been consistent in recent years in Albania [47]. This finding contrasts with findings from a number of other studies. In Italy among HCWs in a large university hospital during the COVID-19 pandemic, influenza vaccination coverage in males was higher than in females for 3 consecutive seasons [49]. Two recent literature reviews of influenza vaccine uptake among HCWs also found that globally influenza vaccine uptake tended to be higher among male HCWs compared to female HCWs [16, 50]. Further studies could help better understand the reasons for sex differences in influenza vaccine coverage among Albanian HCWs.

We found significant differences in uptake of influenza and COVID-19 vaccine among HCWs by hospital. These differences may reflect differences in the vaccination campaigns in the hospitals, accessibility to vaccine, or other factors. Better understanding the reasons behind these differences, and learning from approaches taken in hospitals with higher vaccine uptake, could help inform future vaccination campaigns targeted at hospital-based HCWs.

### Limitations

Our study is subject to several limitations. First, we enrolled participants through a convenience sample, which may have introduced self-selection bias, which may have impacted measures of effect and may limit the generalizability of the study results. While nearly one-third of HCWs in the 3 hospitals participated in the study, we do not know to what extent our study participants were representative of all HCWs in all 3 hospitals. In addition, we could not compare the demographic and occupational distribution of HCWs in our study to those of all HCWs in Albania; therefore, it is unclear if participants in our study were generally representative of hospital-based HCWs in the country. Furthermore, our convenience sample may have overrepresented HCWs who were more motivated to participate, potentially biasing the findings toward HCWs who were more likely to be vaccinated against COVID-19 and influenza. While mandatory vaccination was in place for the COVID-19 primary vaccine series, booster doses and influenza vaccine were not mandatory, which likely impacts the coverage rate and factors associated with vaccination uptake. Additionally, we did not evaluate the correlation between receiving a COVID-19 and an influenza vaccine; future analyses should consider this association, which could inform decisions about coadministration of the 2 vaccines. Finally, because we relied on self-report of influenza vaccination status, it is possible that influenza vaccination status may have been misclassified. Finally, we cannot exclude residual and unmeasured confounding as a partial explanation of our findings.

## CONCLUSIONS

We found that during the 2022–2023 winter season in Albania, only one-fifth of HCWs had received a COVID-19 booster dose; older HCWs, male HCWs, and physicians were more likely to have received COVID-19 PVS and booster vaccines. Fewer than one quarter of HCWs received an influenza vaccine; older HCWs and female HCWs were more likely to receive an influenza vaccine. Confidence in the safety and effectivity of vaccines was positively associated with receiving both COVID-19 and influenza vaccines. Trying to better understand why certain subpopulations of Albanian HCWs were less likely to get vaccinated against COVID-19 and influenza is crucial to inform future vaccine campaigns. Emphasizing the safety and effectiveness of COVID-19 and influenza vaccines, particularly among women of reproductive age, and the continued impact of these two vaccines on morbidity and mortality from both viruses, could help increase vaccine uptake among HCWs in Albania in the future. Future research should explore interventions to address vaccine hesitancy, improve accessibility, and evaluate the effectiveness of tailored communication strategies, targeted educational initiatives, and workplace-based peer education campaigns to promote vaccine uptake.

## Notes

***Acknowledgments.*** The authors thank the healthcare workers in the 3 Albanian hospitals and especially participants in our study. We appreciate the work of the Institute of Public Health team and teams from the 3 hospitals of Tirana, Fier, and Durres. We thank our colleague Kathryn Lafond from U.S. Centers for Disease Control and Prevention (CDC) for her helpful contributions to this work, and we thank Kerry Gallo from the Task Force for Global Health for her help editing our article. We thank Richard Pebody from WHO/European regional Office and Iria Preza from the WHO Country Office–Albania. We thank Ledia Agolli (Southeast European Center for Surveillance and Control of Infectious Diseases, Albania).

***Ethical clearance.*** The study was approved by the Ethics Review Board of the World Health Organization (reference number CERC.0097A) and the Albanian Institute of Public Health (reference number 156). The United States determined that this study met the requirements of public health surveillance as defined in 45 Code of Federal Regulations 46.102(l) (2) (CDC reference number 0900f3eb81ce0ede). This study was registered with ClinicalTrials.gov (NCT04811391). All participants provided written informed consent.

***Financial support.*** This work was supported by funding from the U.S. Centers for Disease Control and Prevention NU51IP000942.
